# Spin doping using transition metal phthalocyanine molecules

**DOI:** 10.1038/ncomms13751

**Published:** 2016-12-12

**Authors:** A. Atxabal, M. Ribeiro, S. Parui, L. Urreta, E. Sagasta, X. Sun, R. Llopis, F. Casanova, L. E. Hueso

**Affiliations:** 1 CIC nanoGUNE, Tolosa Hiribidea 76, 20018 Donostia-San Sebastian, Spain; 2 CAS Center for Excellence in Nanoscience, Key Laboratory of Nanosystem and Hierarchical Fabrication, National Center for Nanoscience and Technology, Beijing 100190, P. R. China; 3 IKERBASQUE, Basque Foundation for Science, 48011 Bilbao, Spain

## Abstract

Molecular spins have become key enablers for exploring magnetic interactions, quantum information processes and many-body effects in metals. Metal-organic molecules, in particular, let the spin state of the core metal ion to be modified according to its organic environment, allowing localized magnetic moments to emerge as functional entities with radically different properties from its simple atomic counterparts. Here, using and preserving the integrity of transition metal phthalocyanine high-spin complexes, we demonstrate the magnetic doping of gold thin films, effectively creating a new ground state. We demonstrate it by electrical transport measurements that are sensitive to the scattering of itinerant electrons with magnetic impurities, such as Kondo effect and weak antilocalization. Our work expands in a simple and powerful way the classes of materials that can be used as magnetic dopants, opening a new channel to couple the wide range of molecular properties with spin phenomena at a functional scale.

## Abstract (web summary)

Molecular magnets are molecules with an inherent non-zero spin that can exhibit magnetic ordering. Here, the authors show that such molecules can change the many-body ground state of nonmagnetic metals at a functional scale with magnetic phthalocyanines.

Metal-organic molecules have been explored for their applications in (opto)electronics^1^,^2^ and spintronics^3^,^4^,^5^,^6^,^7^,^8^. Metal phthalocyanine (MPc) complexes in particular assume a prominent position among the organic semiconductors for their structural simplicity, chemical and thermal stability, compatibility with current large-scale plastic electronics and reliability^9^,^10^,^11^. The coordination of the metal core ion determines its oxidation state and enables localized magnetic moments to emerge in the molecule. The planar structure of the MPc molecule screens the in-plane metal core orbitals while allowing the *π* orbitals to interact out-of-plane. Accordingly, when MPc molecules are placed on a metallic substrate, the resulting magnetic moment is short-ranged and typically requires localized techniques, such as scanning tunneling microscopy, to study its inherent local spin physics^11^,^12^. However, an electronic transport measurement through the metal would provide insight into the new many-body quantum state arising because of the magnetic doping and the interaction of the metal with the molecular spins.

Magnetic doping has witnessed a surge of interest in the past decade^13^, benefiting from advances in epitaxy^14^ and deposition technologies^15^. The search for multifunctional systems with combined properties of the host material and of the magnetic impurity leads to the revival of the field. Recently, self-assembled metal-core molecular monolayers have been explored for the magnetic doping of metals. By destroying the organic framework with an energetic metal layer deposition, the magnetic metal-core impurities are dissolved into the metal to form a magnetic dilute alloy. This molecular carrier approach necessarily relies on magnetic transition metal core ions, and does not benefit from the physical properties of the molecules^4^,^16^, although avoiding the clustering of dopants.

In the following we demonstrate that the intrinsic spin contained in a metal-organic molecule can be used at a functional scale as a magnetic dopant in a metal, following an approach that preserves the integrity of the molecule. This demonstration shows the potential of combining molecular entities with conventional metals for exploring novel magnetic phenomena and materials. We primarily demonstrate our concept using copper (II) phthalocyanine (CuPc), a paramagnetic metal-organic molecule with spin angular momentum *S*=1/2 arising from the oxidation state of the core metal ion, Cu^2+^ (ref. 1). Using gold as host metal and varying the amount of deposited CuPc, we detect and tune the anomalous resistivity increase for low temperatures and determine the Kondo temperature^17^,^18^,^19^, *T*_K_, of the molecular magnetic impurities in gold. By measuring the magnetoconductance at low temperatures for different CuPc thicknesses, we determine that the phase coherence length, *l*_*ϕ*_, decreases for higher thicknesses of molecular layer, indicating an increasing suppression of the coherent backscattering^20^ of the conduction electrons in gold. We complement this study using cobalt (II) phthalocyanine (CoPc), and perform control experiments with nickel (II) phthalocyanine (NiPc), phthalocyanine (Pc) and Cu.

## Results

### Device scheme and fabrication

**Figure 1:**
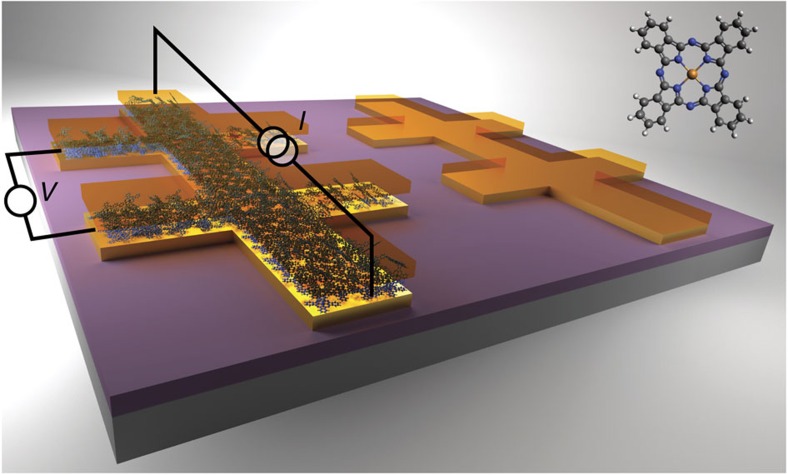
Schematic of the chip carrier. Chip carrier with two Hall bars. The left bar refers to the Au/MPc/Au stack, and the right bar to the bare-gold control reference. The reference device provided the baseline to assess the gold quality of the different devices fabricated. The excitation current is injected along the bar, with two sensing contacts measuring the voltage drop. Inset: molecular structure of the transition metal M=Cu, Co, Ni (II) phthalocyanine complex. Transition metal (golden) ligands to nitrogen atoms (blue) result in total spin angular momentum of *S*=½, ½ and 0 for the cases of CuPc, CoPc and NiPc, respectively. Carbon atoms are represented in grey and hydrogen atoms in white.

The evaporation of MPcs enables the full *in situ* large area fabrication of metal/molecule/metal stacks in ultra-high vacuum conditions (see Methods). Our samples consist of two Hall bars, one of Au (100 Å)/MPc (*t*)/Au (100 Å), where *t* is the nominal thickness of the MPc, and the other is a bare-gold control reference with nominal thickness of 200 Å (see Fig. 1). In both cases the deposition results in a continuous gold film, with likely penetration between the top and bottom layer of gold for the Au/MPc/Au stack. The epitaxial growth and preferential orientation of MPcs depend strongly on the surface where they are being grown, total thickness, temperature of deposition and core metal ion^21^,^22^. For the particular case of CuPc, the high surface energy of gold together with the planar structure of the molecule results in the first CuPc monolayer adsorbing flat to the metal surface^21^,^22^. In the range of molecule thicknesses evaporated in this work, CuPc thin films grown on Au(110) and Au(100) surfaces are known to exhibit order-disorder-order behaviour, with transitions occurring in less than one monolayer coverage, requiring several molecular layers before achieving order as several tens of nanometer thick bulk-like films^21^,^22^. Merging this likely picture with the fact that gold density is larger than the density of the molecular film, we expect gold to penetrate through the molecular thin film during the evaporation process. The penetration provides the mechanism to effectively modulate the doping with molecular thickness. The top gold layer in the Au/CuPc/Au stack was found to be critical in this study, as without it there would not be enough interaction between the molecular spins and the gold conduction electrons (see Supplementary Fig. 1 and Supplementary Note 1) to obtain a clear signal. Complementarily, Raman spectrometry of the stacks confirms that the molecules maintain their structural integrity after the gentle top metal evaporation at a rate of 0.1 Å s^−1^ by exhibiting all the vibrational modes characteristic of the CuPc molecule (see Supplementary Fig. 2, Supplementary Table 1 and Supplementary Note 2). The inset of Fig. 1 depicts the planar MPc molecule, C_32_H_16_MN_8_, with the core transition metal ion, M=Cu, Co, Ni, covalently bonded to the four nearest neighbour nitrogen atoms.

### Electrical characterization of Au/CuPc/Au thin films

**Figure 2:**
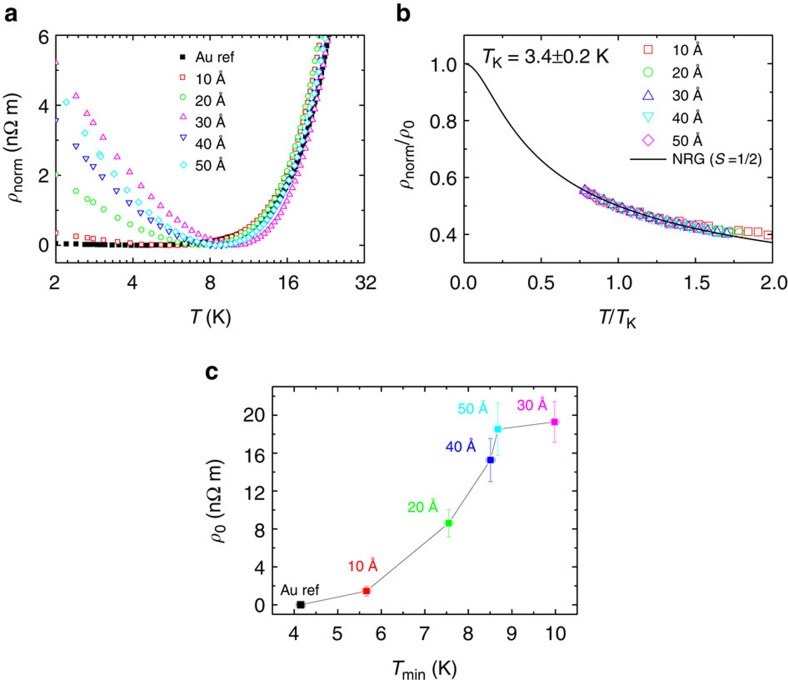
Kondo effect for different molecular thicknesses of CuPc. (**a**) Normalized resistivity versus temperature in log-2 scale. Open symbols correspond to CuPc nominal thicknesses of 10, 20, 30, 40 and 50 Å. Black solid line corresponds to the 99.99% pure 200 Å thick gold reference film. (**b**) Universal Kondo plot of the normalized resistivity divided by respective residual resistivity, 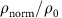, against temperature normalized to the Kondo temperature, 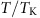 , for all thicknesses. Solid line represents the exact numerical renormalization group (NRG) solution predicted for a *S*=1/2 system of noninteracting diluted magnetic impurities. *T*_K_ was determined to be 3.4±0.2 K. (**c**) *ρ*_0_ as a function of *T*_min_ for the different CuPc thicknesses. Error bars were determined from the s.d. of the fittings.

To determine the contribution of the CuPc magnetic impurities to the electronic transport, the resistivity of five samples with *t* of 10, 20, 30, 40 and 50 Å, together with respective gold references, were measured from room temperature down to 2 K without external magnetic fields (see Methods and Supplementary Fig. 3, Supplementary Table 2 and Supplementary Note 3). These samples display a minimum in the resistivity, *ρ*_min_, at a particular temperature, *T*_min_. In order to make the resistivity measurements directly comparable, we performed a resistivity normalization^4^. The normalized resistivity *ρ*_norm_ is calculated as 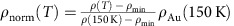, where *ρ*_Au_(150 K) is the resistivity at *T*=150 K of the gold reference sample, and *ρ*(150 K) the resistivity at *T*=150 K of the Au/CuPc/Au stack. Figure 2a shows the normalized resistivity as a function of temperature in log-2 scale for the CuPc samples and the gold reference. The sheet resistance (*R*_s_) and residual resistivity ratio (*RRR*) for each CuPc sample is included in the Supplementary Table 2 and Supplementary Note 3. The resistivity of the gold reference samples exhibits a *T*^2^ dependence at high temperatures, confirming the good quality of the deposited gold^23^. This resistivity decreases monotonically down to 4.0±0.1 K, below which there is a small upturn that could be due to residual magnetic impurities or electron–electron interaction. On the contrary, below *T*_min_ the resistivity of the CuPc samples increases monotonically down to 2 K for all thicknesses. *T*_min_ shifts towards higher temperatures whereas the resistivity upturn increases in magnitude roughly proportionally with the molecular thickness, although with some deviations that can be explained by the dispersion of the deposition conditions. The observed dependence of *T*_min_ and of the resistivity with temperature and molecular thickness points out to the presence of Kondo effect^24^. This effect arises from the antiferromagnetic exchange coupling of the magnetic moments of the impurities with the itinerant electrons of the host material that leads to a characteristic logarithmic increase of the resistivity at low temperatures^25^. An increment in the number of magnetic impurities leads to a larger normalized residual resistivity at *T*=0 K, *ρ*_0_, as well as *T*_min_ (ref. 26). One of the main parameters characterizing this effect is *T*_K_, related to the exchange interaction coupling and hence independent of the concentration of impurities^26^. Following the numerical renormalization group theory for *S*=1/2 systems^27^ (see Supplementary Note 4), the *T*_K_ for CuPc was determined to be 3.4±0.2 K. This temperature readout might be lower than the one obtained via a scanning tunneling microscopy approach, and is related to the broadening of the coupling of the molecular spins to the conduction electrons following our large ensemble diffusive transport approach.

Figure 2b shows the fitting of the experimental data for temperatures below *T*_min_ to numerical renormalization group theory stated before^26^,^27^ (see Supplementary Note 4). The data are displayed in the universal Kondo plot, 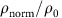 as a function of 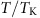, with the contribution to the normalized resistivity from each reference subtracted from the respective CuPc sample for *T<T*_min_. This fitting is a direct proof of the presence of the Kondo effect and subsequently indicates that the molecular impurities retain their spin state. The metal ion in the CuPc molecule behaves as a magnetic centre with *S*=1/2. Figure 2c summarizes the information obtained from the fittings. *ρ*_0_ and *T*_min_ are roughly proportional, showing both the robustness of the continuous doping and of our analysis.

**Figure 3:**
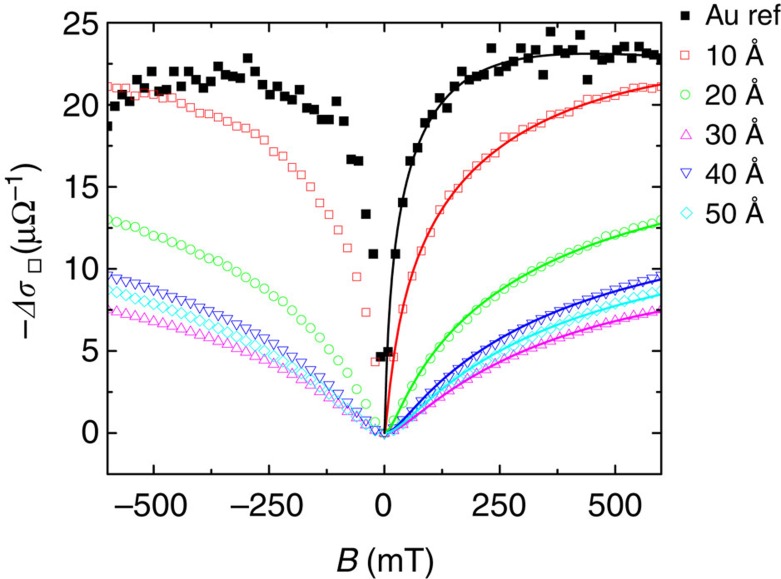
Magnetoconductance measurements at 4 K. Open symbols correspond to CuPc nominal thicknesses of 10, 20, 30, 40 and 50 Å. Solid squares corresponds to the 200 Å thick gold reference film. Solid lines are fits to the Hikami, Larkin and Nagaoka (HLN) theory (equation (1)) for the magnetoconductance of thin films. Extracted phase coherence lengths are 650, 284, 127, 80, 94 and 86 nm for CuPc thicknesses of 0, 10, 20, 30, 40 and 50 Å, respectively.

Magnetic impurities in disordered metals with strong spin–orbit coupling suppress the constructive interference of the back-scattered electrons known as weak antilocalization^20^,^28^,^29^. This effect decreases the phase coherent length, *l*_*ϕ*_, leading to a widening of the magnetoconductance^20^. The determination of *l*_*ϕ*_ for the different Au/CuPc/Au samples is then a complementary evidence for the presence of the magnetic impurities in the metal. Figure 3 shows the magnetoconductance, 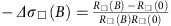, at 4 K for the CuPc samples and their bare-gold control references. The gold deposition was optimized such that both the CuPc samples and bare control references were disordered enough to exhibit weak antilocalization (see Supplementary Fig. 4 and Supplementary Note 3). The observed widening for increasing CuPc thicknesses matches qualitatively the behaviour observed for the dependence of the incremental resistivity with increasing thickness. *l*_*ϕ*_ was determined by fitting the experimental data to the theory of Hikami, Larkin and Nagaoka^30^ (HLN) for the magnetoconductance of thin films:


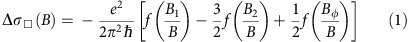


with 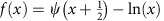, being 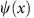 the digamma function, and *B*_1_, *B*_2_ and *B*_*ϕ*_ the characteristic magnetic fields, defined as 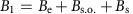, 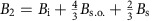 and 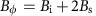, where (e) corresponds to the elastic, (i) inelastic, (s.o.) spin–orbit and (s) magnetic scattering mechanisms. Assuming that the inelastic scattering depends only on temperature, we attribute the decrease in *l*_*ϕ*_ to the contribution introduced by the magnetic impurities. The scaling of the resistivity with the thickness of CuPc is assumed to be mainly from interface roughness and increased disorder (see Supplementary Note 3). From the fittings and using 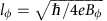, where *ħ* is the reduced Planck constant, *e* the electron charge and *B*_*ϕ*_ the characteristic magnetic field for dephasing mechanisms, we can obtain *l*_*ϕ*_ for each thickness of molecular deposition. The phase coherence length roughly decreases with increasing thickness, in excellent agreement with the increase of the resistivity upturn in the temperature dependence of the resistivity. These low-temperature magnetotransport measurements confirm the doping of gold with molecular magnetic impurities.

### Control experiments

**Figure 4:**
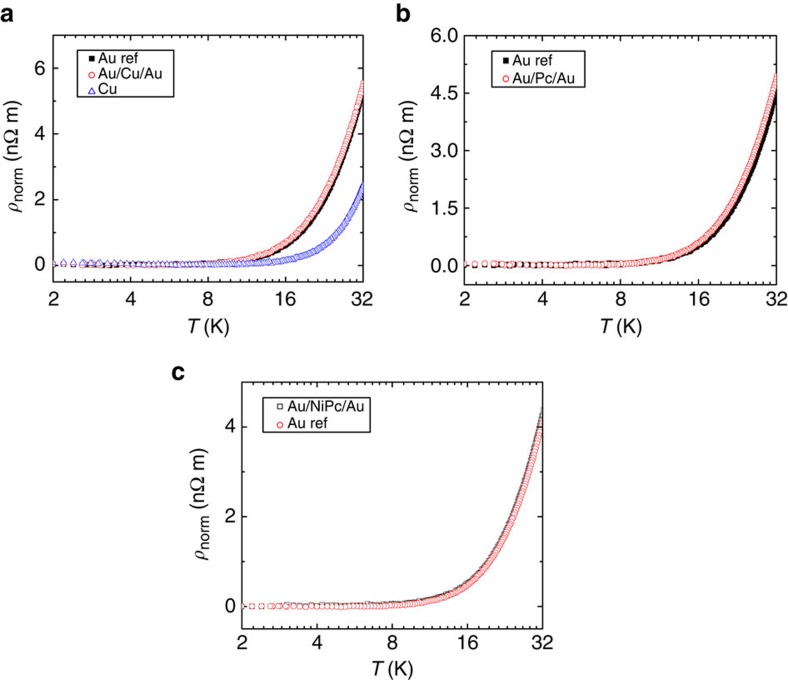
Normalized resistivity versus temperature for control samples of gold with impurities. Gold references are 200Å thick, and temperature axes are in log-2 scale. (**a**) Gold reference sample (black solid squares) versus 200 Å gold sample with 20 Å of copper (red open circles) and 200 Å copper sample (blue open triangles). (**b**) Gold reference sample (black solid squares) and 200 Å gold sample with 50 Å of Pc (red open circles). (**c**) Gold reference sample (black solid squares) versus 200 Å gold sample with 50 Å of NiPc (red open circles).

Up to now, we have demonstrated that CuPc molecules sandwiched in between gold films act as magnetic impurities. This is an exciting result as virtually any transition metal in which a spin state can be stabilized by molecular coordination could be used as a magnetic dopant. However, we have not unambiguously demonstrated that the molecular spins are acting as magnetic impurities. In the first place, and although Raman measurements confirm molecular structural integrity (Supplementary Fig. 2, Supplementary Table 1 and Supplementary Note 2), some of the molecules could be destroyed during the fabrication process and release copper impurities that carry a spin *S*=1/2. To the best of our knowledge, there is not any experimental demonstration of Kondo effect with copper impurities in gold. Even so we have performed several crosscheck experiments. The deposition of copper metal (Fig. 4a) or empty phthalocyanine (Pc) (Fig. 4b) instead of CuPc molecules does not give an upturn in the resistivity of the gold films. These control experiments strongly suggest that the molecular metallic centres play a decisive role for the magnetic interactions and that any eventual disorder created by the molecules has a negligible effect in the electronic transport properties. We have also repeated our experiments with nickel (II) phthalocyanine (NiPc) (Fig. 4c). Nickel is a ferromagnetic transition metal with spin angular momentum *S*=1 in its ground state, whereas NiPc has no net magnetic moment^12^,^31^. Although we use a 5 nm thick NiPc film, the temperature dependence of the resistivity does not present any upturn at low temperature, and this goes in line with the expectation that no upturn should be seen for systems with no net magnetic moment.

**Figure 5:**
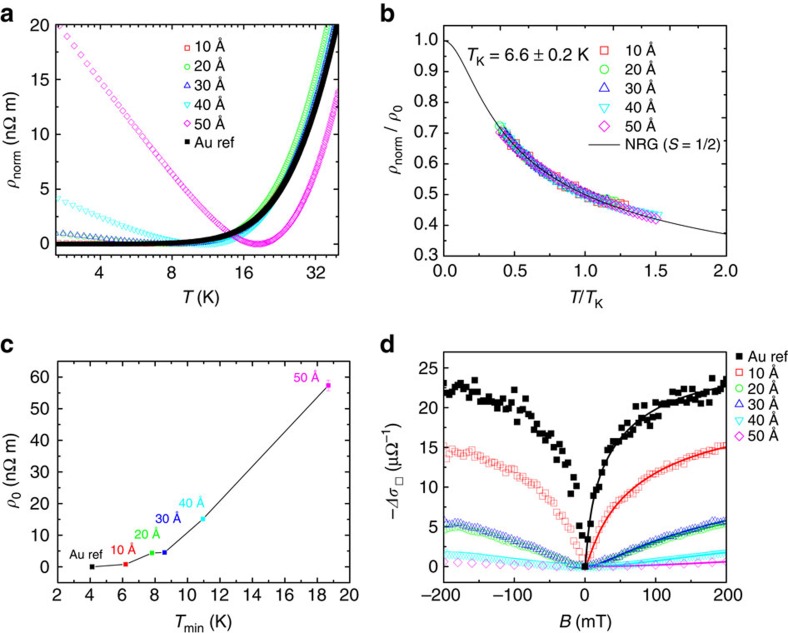
Kondo effect and magnetoconductance measurements at 4 K for samples with different molecular thicknesses of CoPc. (**a**) Normalized resistivity versus temperature in log-2 scale. Open symbols correspond to CoPc nominal thicknesses of 10, 20, 30, 40 and 50 Å. Black solid squares corresponds to the 99.99% pure 200 Å thick bare-gold film. (**b**) Universal Kondo plot of the normalized resistivity divided by respective residual resistivity, 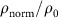, against normalized temperature, 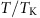, for all thicknesses. Solid line represents the exact numerical renormalization group (NRG)^26^,^27^ solution predicted for a *S*=1/2 system of diluted magnetic impurities. *T*_K_ was determined to be 6.6±0.2 K. (**c**) Determined *ρ*_0_ as a function of *T*_min_ for the different CoPc thickness. Error bars were determined from the s.d. of the fittings. (**d**) Magnetoconductance at 4 K for CoPc nominal thicknesses of 10, 20, 30, 40 and 50 Å. Solid black squares corresponds to the 200 Å thick gold reference film. Solid lines are fits to the Hikami, Larkin and Nagaoka (HLN) theory^30^ for the magnetoconductance of thin films. The phase coherence lengths are determined to be 645, 272, 102, 106, 59 and 40 nm for CoPc thicknesses of 0, 10, 20, 30, 40 and 50 Å, respectively.

We have extended our experiments with another related molecule, cobalt (II) phthalocyanine (CoPc) (Fig. 5), for obtaining a better insight into the doping mechanism. The magnetic ground state of cobalt, which is again a ferromagnetic metal, is *S*=3/2. However, in the CoPc molecule, and due to the nitrogen covalent bonds, the cobalt spin state is *S*=1/2 (ref. 31). In the Au/CoPc/Au sample we observe a clear upturn in the low temperature resistivity (Fig. 5a) and a change in the magnetoconductance (Fig. 5d), clearly indicating that the molecular magnetic impurities are leading to the creation of a Kondo state. The experimental results are indeed very similar to those obtained with doping using CuPc molecules. The fittings to the temperature dependence of the resistivity of the CoPc samples using a *S*=1/2 model (see Supplementary Note 4) yield *T*_K_=6.6±0.2 K (Fig. 5b). This result comes in line with the *T*_K_ of 6.8±0.2 K reported in ref. 3 for a *S*=1/2 model of cobalt impurities in gold. The slightly higher *T*_K_ for CoPc with respect to CuPc indicates a stronger coupling of the magnetic impurities to the conduction electrons in gold. Moreover, Fig. 5c demonstrates again proportionality between *ρ*_0_ and *T*_min_, as expected from Kondo theory. The sheet resistance and residual resistivity ratio (*RRR*) for each CoPc sample is included in the Supplementary Table 3.

**Figure 6:**
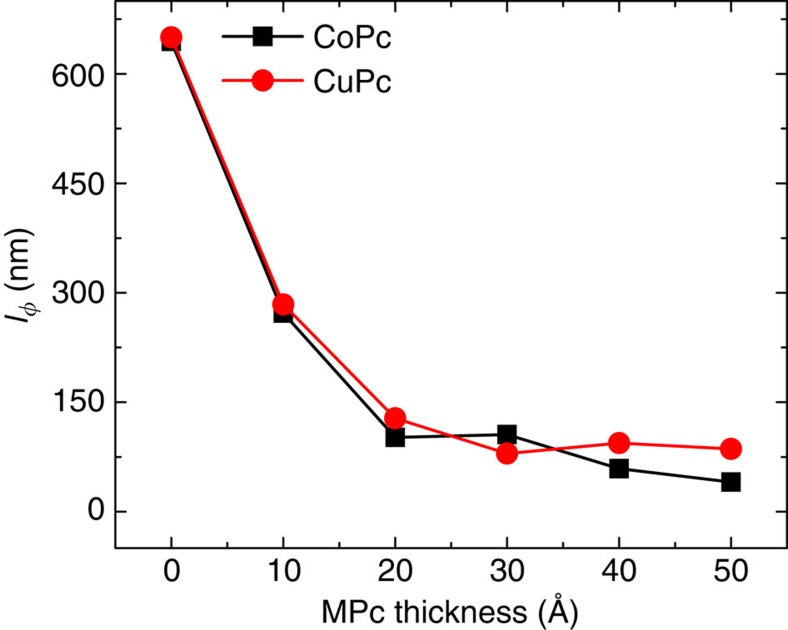
Phase coherence length values. Comparison of phase coherence length at 4 K extracted from fitting the data of Fig. 3 to equation (1) as a function of molecule thickness for samples with CuPc (red solid circles) and from Fig. 5d for samples with CoPc (black solid squares) as impurity. The error bars determined from the s.d. of the fittings are smaller than the symbol size.

Figure 6 summarizes the phase coherence lengths obtained from the HLN fittings (equation (1)) at 4 K for both CuPc and CoPc. The similarity between the two curves, both in the absolute values and in their dependence with the molecular layer thickness, indicates that the magnetic centres in CoPc and CuPc contribute similarly to the scattering of the conduction electrons. This similarity is perfectly logical considering that both CuPc and CoPc carry the same spin state. These results add yet another independent confirmation that copper and cobalt retain their oxidation state, and their bonds to the neighbouring nitrogen atoms are preserved.

## Discussion

In conclusion, we have demonstrated the doping of a gold metal thin film with CuPc and CoPc molecular magnetic impurities. Using a simple device architecture and varying the thickness of the deposited MPc, we modulate the magnetic interaction and extend it from a molecular level to a functional scale. Measuring the metal we are able to sense the molecular magnetism. Metal-organic molecules, being versatile for the incorporation of transition metals (magnetic and nonmagnetic), suggest wider limits for the range of materials that can be used as magnetic dopants. As long as a molecular spin state can be stabilized in the metal host, transition metal free organic radicals, such as tris(2,4,6-trichlorophenyl)methyl, can also be sought for molecular spin doping, as well as molecules with higher spin states. Moreover, by extending the spin state, metal organic molecules might be enhancing the interaction of the magnetic impurities and the host material, giving rise to a stronger coupling. Because of the intrinsic properties of organic molecules, such as photosensitivity, molecular spin doping can be used as a new platform to combine magnetic and optical effects. This can be extended to the wide range of properties that organic molecules exhibit. Device applications that envision the coupling of the molecular properties to the spin state of the molecule can lead to new approaches to explore quantum information at a functional scale.

## Methods

### Device fabrication

The devices were fabricated on 10 × 10 mm^2^ Si/SiO_2_ substrates, where before gold evaporation an adhesion layer of 30 Å of aluminum was deposited by effusion cell evaporation and plasma oxidized for 6 min. A layer of 100 Å of gold was then deposited by e-beam evaporation using shadow masking techniques for the simultaneous patterning of two equal Hall bars on the chip carrier at a rate of 0.1 Å s^−1^. Without breaking vacuum, 10 to 50 Å of high purity (>99%) copper (II) phthalocyanine acquired from a commercial supplier (Sigma-Aldrich) were evaporated at a rate of 0.1 Å s^−1^ on one of the Hall bars using shadow masking. Again, *in situ*, 100 Å of gold were deposited on top of the two Hall bars at the low rate of 0.1 Å s^−1^. All the evaporations were performed *in situ* and in ultrahigh vacuum conditions (10^−8^–10^−9^ mbar), with the reported thickness values calibrated with depositions onto Si/SiO_2_ substrates.

### Electrical characterization

Transport characterization was carried out with standard four-probe measurements, using Keithley 6221 nanovoltmeter and Keithley 2182 DC current source, with an excitation current of 10 μA, in a Quantum Design physical property measurement system. A channel width *W* of 1 mm and a channel length *L* of 2.25 mm were considered for the purpose of normalized resistivity and magnetoconductance calculations.

### Data availability

The data that support these findings are available from the corresponding author (L.E.H.) upon reasonable request.

## Additional information

**How to cite this article:** Atxabal, A. *et al*. Spin doping using transition metal phthalocyanine molecules. *Nat. Commun.* **7**, 13751 doi: 10.1038/ncomms13751 (2016).

**Publisher's note**: Springer Nature remains neutral with regard to jurisdictional claims in published maps and institutional affiliations.

## Supplementary Material

Supplementary Information Supplementary Figures, Supplementary Tables, Supplementary Notes and Supplementary References
