# Supplementary material for: Spin doping using transition metal phthalocyanine molecules
Source: Nat Commun. 2016 Dec 12;7:13751. doi: 10.1038/ncomms13751 (PMC5159905; doi:10.1038/ncomms13751)
Supplement: Supplementary Information — Supplementary Figures, Supplementary Tables, Supplementary Notes and Supplementary References [file ncomms13751-s1.pdf]

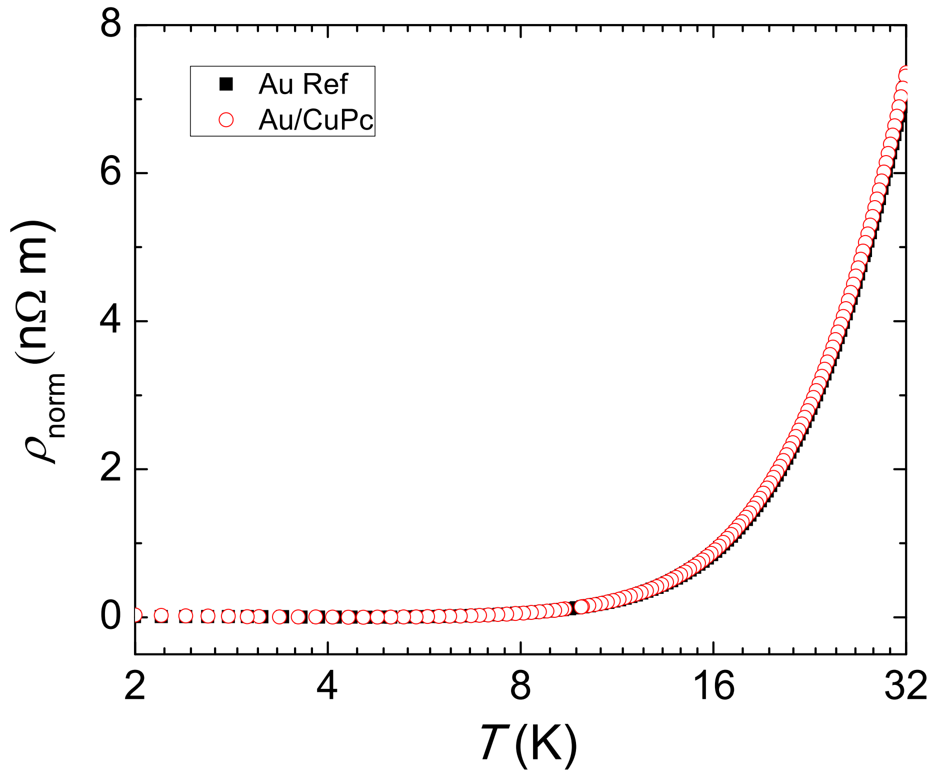

Supplementary Figure 1| Normalized resistivity versus temperature in log-2 scale. Normalized resistivity for 100 Å-thick gold reference sample (black solid squares) and 100 Å gold sample with 50 Å of CuPc (red open circles).

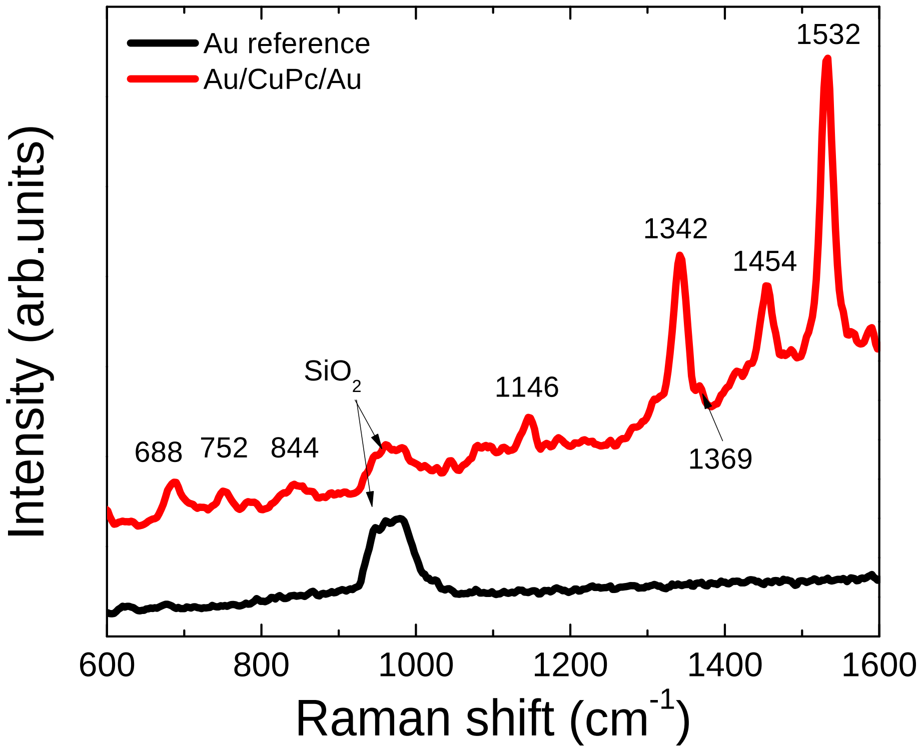

Supplementary Figure 2| Raman spectra for Au/CuPc/Au and gold reference sample. Raman spectra for 200 Å-thick gold reference sample (black solid line) and 200 Å gold sample sandwiched with 50 Å of CuPc (red solid line).

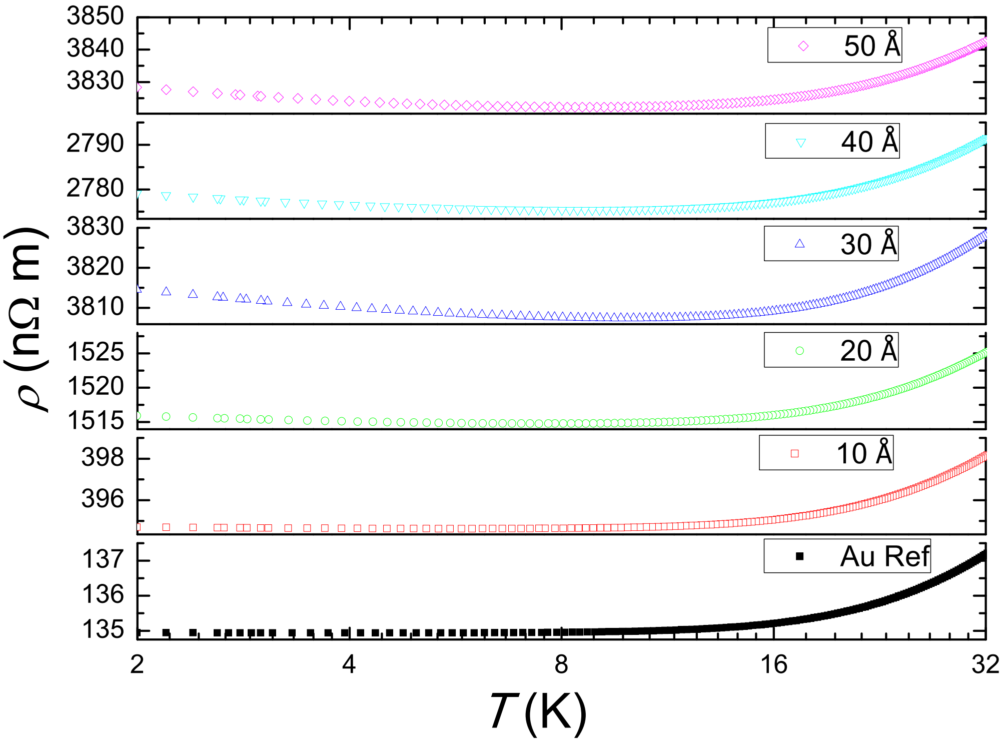

Supplementary Figure 3| Resistivity versus temperature in log-2 scale. As measured resistivity versus temperature in log-2 scale for CuPc nominal thicknesses of 10, 20, 30, 40 and 50 Å in Au(100 Å)/CuPc/Au(100 Å) device structure. Black solid squares correspond to the 99.99% pure 200 Å thick bare-gold film.

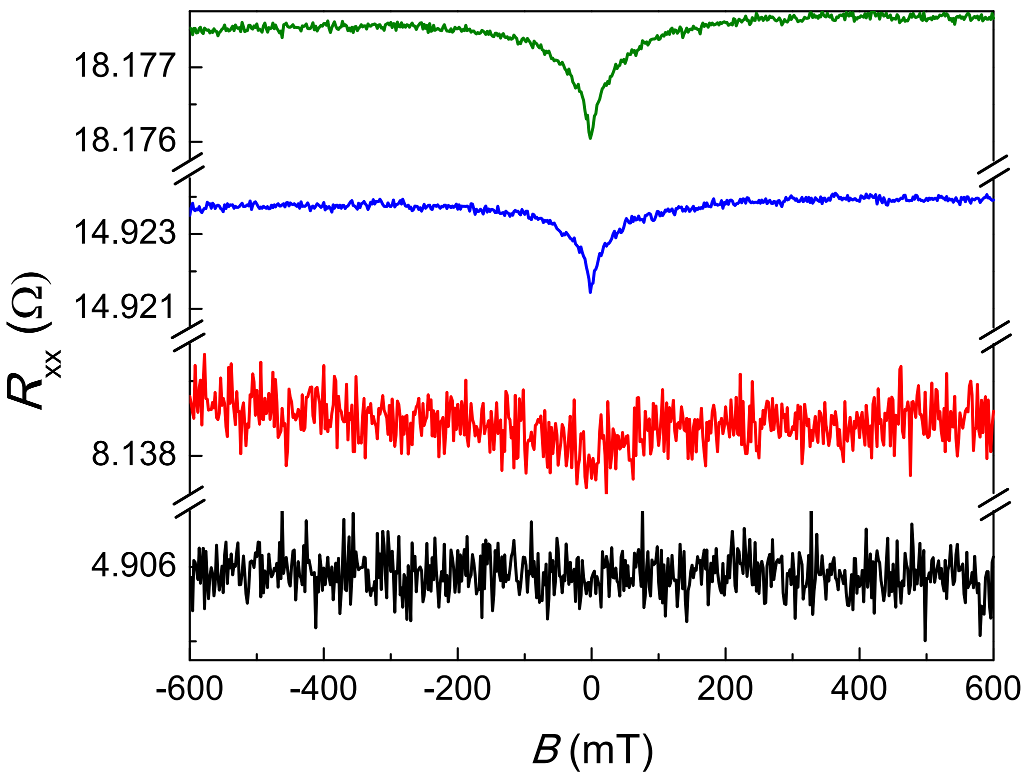

Supplementary Figure 4| Resistance of different gold films versus the perpendicular magnetic field. Resistance of four different reference samples with equivalent geometry versus the applied magnetic field perpendicular to the sample at 4 K.

Supplementary Table 1| Determined peak frequencies for CuPc Raman spectra in comparison with DFT predictions[1].

**Experimental CuPc (cm^{-1})**	**DFT CuPc (cm^{-1})**	**Vibrational mode description**
688±1	674	In plane full symmetric non-metal bound N-M stretch and outer ring stretches
752±1	737	In plane ring symmetric N-M stretch
844±1	830	In plane full symmetric N-M stretch
Masked by SiO_2	938	In plane diagonal symmetric N-M-N bend and N-M stretch
	1098	In plane diagonal symmetric N-M-N bend
1146±1	1133	In plane ring symmetric and outer rings breathing
	1193	In plane diagonal symmetric N-M-N bend
	1290	In plane diagonal symmetric outer ring rotations
1342±1	1343	In plane full symmetric N-C stretch and ring C-C stretch
1369±1	1369	In plane ring symmetric outer ring C-C stretch
	1438	In plane ring symmetric outer ring C-C stretch
1454±1	1439	In plane diagonal symmetric non-metal bound N-C stretch
1532±1	1545	In plane ring symmetric non-metal bound N-C stretch

Supplementary Table 2| Sheet resistance (R_s), resistivity (ρ) of CuPc samples at 2, 150 and 300 K and residual resistivity ratio (*RRR*) for the various nominal molecular thicknesses.

CuPc thickness (Å)	R_s (Ω)	ρ (nΩ·m), 2K		ρ (nΩ·m), 150K		ρ (nΩ·m), 300 K		RRR	
	Sample	Sample	Ref.	Sample	Ref.	Sample	Ref.	Sample	Ref.
**10**	21.6 ±0.1	390 ±20	158 ±8	450 ±20	184.6 ±0.1	475 ±9	210 ±10	1.20 ±0.06	1.36 ±0.07
**20**	81.1 ±0.1	1520 ±70	162 ±8	1620 ±80	188.8 ±0.1	1700 ±90	220 ±10	1.15 ±0.06	1.36 ±0.07
**30**	201.8 ±0.1	3800 ±200	142 ±7	4000 ±200	167.4 ±0.1	4300 ±200	200 ±10	1.13 ±0.06	1.39 ±0.07
**40**	147.0 ±0.1	2800 ±100	135 ±7	2900 ±100	160.0 ±0.1	3100 ±200	190 ±9	1.13 ±0.06	1.40 ±0.07
**50**	201.5 ±0.1	3800 ±200	133 ±7	4000 ±200	157.6 ±0.1	4200 ±200	186 ±2	1.12 ±0.06	1.41 ±0.07

Supplementary Table 3| Sheet resistance (R_s), resistivity (ρ) of CoPc samples at 2, 150 and 300 K and residual resistivity ratio (*RRR*) for the various nominal molecular thicknesses.

CoPc thickness (Å)	R_s (Ω)	ρ (nΩ·m), 2K		ρ (nΩ·m), 150K		ρ (nΩ·m), 300 K		RRR	
	Sample	Sample	Ref.	Sample	Ref.	Sample	Ref.	Sample	Ref.
**10**	21.6 ±0.1	390 ±20	158 ±8	450 ±20	185 ±9	480 ±20	220 ±10	1.20 ±0.07	1.36 ±0.07
**20**	81.1 ±0.1	1520 ±80	162 ±8	1620 ±80	189 ±9	1750 ±90	220 ±10	1.15 ±0.06	1.36 ±0.07
**30**	201.8 ±0.1	3800 ±200	142 ±7	4036.0 ±0.1	167 ±8	4300 ±200	200 ±10	1.130 ±0.06	1.39 ±0.07
**40**	147.0 ±0.1	2800 ±100	135 ±7	2900 ±100	160 ±8	3100 ±200	190 ±10	1.13 ±0.06	1.40 ±0.07
**50**	201.5 ±0.1	3800 ±200	133 ±7	4000 ±200	158 ±8	4300 ±200	190 ±10	1.12 ±0.06	1.41 ±0.07

Supplementary Note 1| Au/CuPc stack without top layer deposition

Supplementary Figure 1 shows the temperature dependence of the normalized resistivity for the Au (100 Å)/CuPc (50 Å) bilayer. No upturn in the resistivity of this sample was observed, leading to the conclusion that top gold layer deposition is necessary to obtain sufficiently enough interaction between molecular spins and gold conduction electrons. This conclusion was the one that drew the attention towards a modulation of the signal with the molecular thickness.

Supplementary Note 2| Raman spectroscopy

Raman spectroscopy measurements of the Au(100 Å)/CuPc(50 Å)/Au(100 Å) and 200 Å-thick gold reference samples were obtained using a confocal Raman spectrometer with a 532 nm laser line at room temperature. Supplementary Figure 2 shows the spectra for the gold reference sample (black solid line), and the Au/CuPc/Au (red solid line). The gold reference spectra exhibits only the vibrational mode associated with the background in the range of frequencies explored. The Au/CuPc/Au shows much richer spectra with all the expected CuPc vibrational modes. The peak frequencies were determined using a Lorentzian least square best fit and compared to the ones determined from DFT calculations[1]. Supplementary Table 1 summarizes this information. Although some of the predicted peaks are not visible, the observed spectra is in line with what is experimentally obtained in other works for CuPc thin films[1].

Supplementary Note 3| Sheet resistance and resistivity

Supplementary Table 2 shows the sheet resistance (R_s), resistivity of CuPc samples at 2, 150 and 300 K, and residual resistivity ratio (*RRR*) for the various molecular thicknesses. Supplementary Table 3 shows the same data for the case of CoPc samples. To benchmark the 200 Å reference gold thin films used in this work, we compare them to high quality epitaxial thin films of gold grown on sapphire (0001) substrates[2]. The reference gold thin films in this work exhibit higher residual resistivity and lower *RRR*, which is a sign of increased disorder. For film thicknesses of 200 Å the reported resistivity for high quality epitaxial thin films is of 20 nΩ m, and *RRR* of approximately 2. The gold reference devices are more resistive (~170 nΩ m) and with *RRR* values of approximately 1.4. Also we added in Supplementary Figure 3 the resistivity versus temperature in log-2 scale without the normalization for the CuPc samples.

Gold deposition settings were optimized for increased disorder, such that the weak-antilocalization signals were more evident and comparable. Supplementary Figure 4 displays the resistance of four 200 Å thick gold reference samples as a function of an applied magnetic field perpendicular to the sample. Samples were grown for different gold deposition settings and calibrated accordingly. Weak anti-localization is observed only for the samples with higher resistance. For the disordered gold reference samples used in this work, the obtained values for the characteristic magnetic field of the spin-orbit scattering mechanism, $B_{s.o.}$ = 0.09 T, match quantitatively those of other works on disordered gold thin films[3].

Supplementary Note 4| Determination of the Kondo temperature and residual resistivity

Following the work of supplementary reference 4, the determination of the Kondo temperature and residual resistivity of the CuPc and CoPc magnetic impurities in gold is based on the Ansatz

$$\rho_{\mathrm{norm}}(T) = \delta + \rho_{\mathrm{m}}(0)\left(1+\left(2^{\frac{1}{\alpha_{\mathrm{s}}}}-1\right)\left(\frac{T}{T_{\mathrm{K}}}\right)^{2}\right)^{-\alpha_{\mathrm{s}}} \qquad (1)$$

where $\rho_{\mathrm{norm}}(T)$is the experimental normalized resistivity, δ an offset term, $\rho_{\mathrm{m}}(0)$ the residual resistivity at T = 0 K, T_{K} the Kondo temperature, and α_{s} an exponent term that reflects the spin model of the magnetic impurity. This model has been demonstrated to match with numerical renormalization group theory for S=1/2, 1 and 3/2 of dilute magnetic moments in metallic hosts[4]. For a spin ½ model, $\alpha_{\mathrm{s}} = 0.21$. At very low temperatures, the contribution of the electron-phonon scattering mechanism is neglected and the electron-electron interactions subtracted using the reference temperature dependence for the same range of temperatures.

Supplementary References

1. Jiang, N. *et al.* Observation of Multiple Vibrational Modes in Ultrahigh Vacuum Tip-Enhanced Raman Spectroscopy Combined with Molecular- Resolution Scanning Tunneling Microscopy. *Nano Lett.* **12,** 5061–5067 (2012).

2. Kastle, G., Boyen, H.-G., Schröder, A., Plettl, A. & Ziemann, P. Size effect of the resistivity of thin epitaxial gold films. *Phys. Rev. B* **70,** 165414–6 (2004).

3. Kawaguti, T. & Fijimori, Y. Magnetoresistance and Inelastic Scattering Time in Thin Films of Silver and Gold in weak Localized regime. *J. Phys. Soc. Japan* **52,** 722–725 (1983).

4. Costi, T. A. *et al.* Kondo Decoherence: Finding the Right Spin Model for Iron Impurities in Gold and Silver. *Phys. Rev. Lett.* **102,** 056802–4 (2009).
